# Thyroid hormone elicits intergenerational epigenetic effects on adult social behavior and fetal brain expression of autism susceptibility genes

**DOI:** 10.3389/fnins.2022.1055116

**Published:** 2022-11-07

**Authors:** Maria Elena Martinez, Julia Patrizia Stohn, Elizabeth M. Mutina, Rayne J. Whitten, Arturo Hernandez

**Affiliations:** ^1^Center for Molecular Medicine, MaineHealth Institute for Research, MaineHealth, Scarborough, ME, United States; ^2^Graduate School for Biomedical Sciences and Engineering, University of Maine, Orono, ME, United States; ^3^Department of Medicine, Tufts University School of Medicine, Boston, MA, United States

**Keywords:** thyroid hormone, autism, social behavior, fetal brain, transgenerational epigenetic

## Abstract

Genetic mutations identified in genome-wide association studies can only explain a small percentage of the cases of complex, highly heritable human conditions, including neurological and neurodevelopmental disorders. This suggests that intergenerational epigenetic effects, possibly triggered by environmental circumstances, may contribute to their etiology. We previously described altered DNA methylation signatures in the sperm of mice that experienced developmental overexposure to thyroid hormones as a result of a genetic defect in hormone clearance (DIO3 deficiency). Here we studied fetal brain gene expression and adult social behavior in genetically normal F2 generation descendants of overexposed mice. The brain of F2 generation E13.5 fetuses exhibited abnormal expression of genes associated with autism in humans, including *Auts2, Disc1, Ldlr*, *Per2*, *Shank3*, *Oxtr*, *Igf1*, *Foxg1*, *Cd38*, *Grid2*, *Nrxn3*, and *Reln*. These abnormal gene expression profiles differed depending on the sex of the exposed ancestor. In the three-chamber social box test, adult F2 generation males manifested significantly decreased interest in social interaction and social novelty, as revealed by decrease total time, distance traveled and time immobile in the area of interaction with novel strangers. F1 generation mice, compared to appropriate controls also exhibited altered profiles in fetal brain gene expression, although these profiles were substantially different to those in the F2 generation. Likewise adult F1 generation mice showed some abnormalities in social behavior that were sexually dimorphic and milder than those in F2 generation mice. Our results indicate that developmental overexposure to thyroid hormone causes intergenerational epigenetic effects impacting social behavior and the expression of autism-related genes during early brain development. Our results open the possibility that altered thyroid hormone states, by eliciting changes in the epigenetic information of the germ line, contribute to the susceptibility and the missing—but heriTables—etiology of complex neurodevelopmental conditions characterized by social deficits, including autism and schizophrenia.

## Introduction

The etiology of human neurodevelopmental disorders characterized by abnormal social behaviors has not been fully elucidated. Conditions such as schizophrenia, autistic spectrum disorders (ASD), and attention-deficit hyperactive disorder (ADHD) are highly heritable (Rommelse et al., 2010; Hallmayer et al., 2011; Colvert et al., 2015; Tick et al., 2016; Blokland et al., 2017; Chou et al., 2017; Sandin et al., 2017; Hilker et al., 2018), but mutations in candidate genes identified in genome-wide association studies (GWAS) can only explain a minority of actual clinical cases. This suggests the existence of other factors that contribute to the “missing heritability” of these complex diseases (Manolio et al., 2009; Zuk et al., 2012; Shen, 2013; van Dongen and Boomsma, 2013; Koch, 2014a; Woo et al., 2017; Escher and Ford, 2020). According mostly to studies in rodents, these factors may include environmental exposures or pathological circumstances in previous generations capable of eliciting alterations in the epigenetic information of the germ line (Goriely and Wilkie, 2010; Koch, 2014b; Prokopuk et al., 2015; Rodgers et al., 2015; Trerotola et al., 2015). These epigenetic alterations can in turn modify the developmental programs in the offspring, causing abnormal phenotypes and modifying susceptibility to disease. These effects can be noted in the next and subsequent generations (Rakyan and Whitelaw, 2003; Anway et al., 2006; Gore et al., 2007; Yeshurun and Hannan, 2019), and different paradigms and experimental models of intergenerational epigenetic effects and transgenerational inheritance have been reported. Pioneer studies by Pembrey et al. (2006) demonstrated that transgenerational epigenetic effects also occur in humans.

Models of environmental exposures in human and rodents have demonstrated intergenerational epigenetic effects involving neurological traits and social behaviors of potential relevance to ASD, ADHD, and schizophrenia (Escher et al., 2021). Grandchildren of women exposed to the synthetic estrogen diethylstilbestrol were at increased risk of developing ADHD (Kioumourtzoglou et al., 2018), and ASD-related neurobehavioral phenotypes have been observed in the children that were exposed *in utero* to the antiepileptioc drug valproic acid (Roullet et al., 2013). Grandchildren of women that smoked during pregnancy also exhibited an increased risk of ASD (Golding et al., 2017). Furthermore, an epigenetic signature has been identified in the sperm of fathers with children with autism (Garrido et al., 2021). Observations in rodent models show that the offspring of pregnant mice exposed to the anesthetic sevofluorane manifested social deficits, and this behavioral abnormality was transmitted for two more generations (Wang et al., 2021). Second and third generation descendants of mice exposed to bisphenol A showed social deficits and aberrant brain expression of social neuropeptides oxytocin and vasopressin (Wolstenholme et al., 2012, 2019; Goldsby et al., 2017; Drobná et al., 2018). Similarly, investigators have observed social deficits in second generation descendants of rats exposed to fungicide vinclozolin or polychlorinated biphenyls (Krishnan et al., 2018, 2019) and in the offspring and second generation descendants of mice exposed to valproic acid (Choi et al., 2016). Thus, environmental exposure can cause intergenerational epigenetic effect on neurological traits of significance to neurodevelopmental disorders.

Alterations in thyroid hormone states can also produce epigenetic effects in subsequent generations. Seminal work by Bakke showed that rats neonatally injected with thyroid hormone or made hypothyroid in adulthood, generated offspring with abnormal phenotypes affecting growth, developmental milestones, and neuroendocrine traits (Bakke et al., 1976, 1977). The occurrence of transgenerational effects of thyroid hormones in humans was recently demonstrated by Anselmo et al. (2019) studying an Azorean population carrying a mutation in the thyroid hormone receptor beta. During pregnancy, women carrying one copy of this mutation develop pituitary resistance to thyroid hormone and hyperthyroidism, affecting the fetus (Anselmo et al., 2004). In adult life, genetically normal children born to these women exhibit altered pituitary sensitivity to thyroid hormones, a trait that is transmitted to two more generations along the paternal line (Anselmo et al., 2019).

We have recently shown transgenerational effects driven by thyroid hormones in a mouse model of thyroid hormone excess secondary to a deficiency in the type 3 deiodinase (DIO3). As DIO3 clears thyroid hormones and is particularly abundant in the pregnant uterus, placenta and developing tissues, including the central nervous system (Hernandez, 2005; Hernandez et al., 2021), *Dio3−/−* mice experience developmental thyrotoxicosis (Hernandez et al., 2006; Martinez and Hernandez, 2021). This insult is associated with DNA hypomethylation in neonatal spermatogonia and alterations in the adult sperm methylome affecting a significant proportion of candidate genes for neurological disease, including ASD and schizophrenia (Martinez et al., 2020). At postnatal day 15 (P15), the F2 generation descendants of *Dio3−/−* mice manifest aberrant patterns of brain gene expression, which was associated with decreased levels of locomotor activity and anxiety-related behaviors (Martinez et al., 2020). However, we have not determined the consequences for social behavior, a phenotype profoundly altered in directly exposed ancestors (Stohn et al., 2018). In addition, the genes differentially expressed in the P15 brain of F2 descendants did not show a strong overlap with those affected by differential methylation in the sperm of exposed ancestors. This raised the possibility that the latter group of genes is differentially expressed at much earlier stages of brain development.

Here we show that as early as embryonic day 13.5, F2 generation descendants of mice that experienced developmental thyrotoxicosis, show altered expression of genes related to ASD and schizophrenia, as well as altered behavior in the three-chamber social box paradigm that resemble those previously described for the exposed ancestors (Stohn et al., 2018). Our results indicate that altered thyroid hormones status may also cause transgenerational epigenetic effects of relevance to early brain development and social behaviors, with implications for the non-genetic inherited etiology of neurodevelopmental disorders in humans.

## Materials and methods

### Experimental mice

As a model of developmental overexposure to thyroid hormone (T3), we used mice genetically deficient in the type 3 deiodinase (DIO3). We have previously described that *Dio3*−/− mice exhibit markedly elevated serum levels of T3 during fetal and early life (Hernandez et al., 2006). All experimental mice were on an outbred CD-1 genetic background to overcome the impaired fertility of *Dio3*−/− mice on inbred genetic backgrounds. The original mutant mouse strain was generated in a 129/SvJ genetic background (Hernandez et al., 2002, 2006) and has been backcrossed on a CD-1 background for more than 12 generations. Due to the genomic imprinting of the *Dio3* gene (Hernandez et al., 2002; Tsai et al., 2002; Martinez et al., 2014), the colony has been maintained for more than 20 generations by crossing wild type males with heterozygous females, so that the heterozygous mice generated are phenotypically normal, as they carry the *Dio3* mutation in the maternal allele, which is already largely suppressed due to genomic imprinting (Hernandez et al., 2002; Tsai et al., 2002). Approximately every six generations, the genetic background of the colony has been refreshed with a wild type CD-1 male purchased from Charles River. To avoid the influence of confounding factors and minimize variability, mothers of fetal and adult experimental animals were mated at 2 months of age and all experimental animals were born before the mother was 5 months old. For experimental fetuses, the morning a vaginal plug was noticed was considered embryonic day 0.5. Adult experimental mice represent only first litters from three to seven different mothers per experimental group, and litter size was limited to 8–12 animals. Mothers of experimental adult mice were isolated before giving birth to raise the pups in the absence of the father and prevent a concurrent pregnancy when nursing the pups. Animals for adult studies were weaned at the age of 3 weeks and caged in groups of three or four until the time of behavioral testing. All mice were maintained on a 12 h light/dark cycle and food and water were provided *ad libitum*. Mice were euthanized by CO_2_ asphyxiation. All experiments were approved by the MaineHealth Institute for Research Institutional Animal Care and Use Committee (IACUC), under current protocol number 2112.

### Gene expression

Whole fetal brains were harvested and immediately frozen on dry ice. Total RNA was extracted using the RNeasy kit from Qiagen (Valencia, CA, USA). Total RNA (1 μg) was reverse transcribed with M-MLV reverse transcriptase in the presence of random decamers (both from Thermo Fisher Scientific, Waltham, MA, USA) at 65°C for 5 min, then 37°C for 50 min. The 20 μl reverse transcription reactions were DNAse treated and diluted by adding 230 μl of RNase free water. An aliquot of each sample was mixed together for an internal standard and diluted fourfold. Real-time PCR reactions were set up in duplicate with gene-specific primers and SYBR Select Master Mix (Thermo Fisher Scientific, Waltham, MA, USA), and run on the CFX Connect from Bio-Rad (Hercules, CA, USA), where they underwent an initial 10 min denaturing step, followed by 36 cycles of a denaturing step (94°C for 30 s) and an annealing/extension step (60°C for 1 min). For each individual sample, expression was corrected by the expression of housekeeping gene *Actb*, which did not exhibit any significant difference in expression between experimental groups (see Section “Results”). Expression data are shown in arbitrary units and represented as fold-change over the mean value in the corresponding control group. The sequences of the primers used for each gene determination are shown in Supplementary Table 1.

### Behavioral tests

Sociability and social novelty were assessed using the three-chambered social box previously described (Moy et al., 2004; Stohn et al., 2018). At weaning, experimental animals of the same litter were housed together in groups of 3 or 4 of the same sex and experimental group. Experimental mice were single caged 24 h before the test, which was performed at 4 months of age. The test apparatus (obtained from ANY-maze, Stoelting, Wood Dale, IL, USA) was a rectangular open box (60 × 40 cm) (Supplementary Figure 1A). Inside the box, two walls 22 cm in height divided it into three compartments of the same size (40 × 20 cm) that were connected by centered openings. The test comprised of three consecutive trials of 10 min each. At the beginning of each test the mouse was placed in the center box of the apparatus and allowed to explore it freely, and in between trials the mouse was returned to its home cage. The first trial allowed the mouse to adapt to the three-chambered box. Time spent in the individual chambers during the initial 10 min adaption trial was evaluated to detect any potential biases that might influence the actual testing. For the second trial, an unfamiliar mouse (1st stranger) matching the sex, age, and genotype of the test mouse, was placed in the stranger cage (a cylindrical, barred cage 7 cm in diameter) located in the right chamber, while the left chamber, remained empty. The bars in the stranger cages were spaced (7 mm), permitting the animals to sniff each other, but otherwise restricted overt physical contact. For the third trial a second stranger was placed in a second stranger cage located in the left chamber, while the first, now a familiar stranger, remained in the stranger cage of the right chamber. Trials were videotaped using the with the ANY-maze™ video tracking system v5.14 (Stoelting, Wood Dale, IL, USA), which allows for automatic recording of various parameters in custom-designed areas, including distance traveled, time spent in each chamber or area, number of exits/entries from/in any given area; and time during which the mouse was mobile or remained still. At the end of the trials the mouse was returned to its home cage permanently and the three-chambered box and cylinders were wiped clean. The chamber in which we placed the first stranger was alternated between tests, but for the purpose of describing the results we refer to the right chamber as the one in which the first stranger was placed and the left chamber as the one in which the second stranger was placed. In these tests, we used a minimum of 12 mice per experimental group. Behavioral data represents mice from two to three different cohorts generated and tested at different dates.

### Statistical analyses

Statistical analyses were performed using the statistical tools of GraphPad Prism 6 (GraphPad Software, Inc., San Diego, CA, USA). A Student’s *t*-test, or a one-way ANOVA followed by Tukey’s test, respectively, was used to determine statistical significance between two or more groups. Statistical significance was defined as *P* < 0.05. Unless otherwise stated, bars or lines represent the mean ± SEM. The distribution of values for most determinations is shown, as values for individual biological samples (individual mice) are plotted.

## Results

### Altered fetal brain gene expression in the F2 generation of thyroid hormone overexposed mice

We have recently described that the sperm DNA of *Dio3−/−* mice, which are overexposed to thyroid hormone during development, exhibit a decreased in methylation in the promoter region of genes associated in humans with susceptibility to neurodevelopmental disorders such as autism and schizophrenia (Martinez et al., 2020). At postnatal day 15 (P15), the brain of mice that had a *Dio3−/−* paternal grandfather (PGF mice) or a *Dio3−/−* paternal grandmother (PGM mice) manifested extensive changes in gene expression, but with few exceptions, the genes affected showed limited overlap to those genes showing hypomethylation in the sperm of ancestors (Martinez et al., 2020).

**FIGURE 1:**
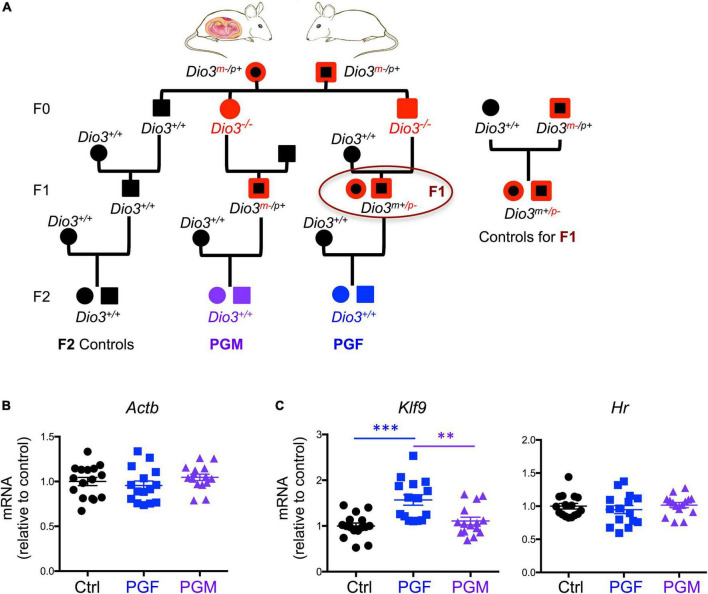
Ancestry tree of the experimental animals and E13.5 brain gene expression of T3 regulated genes. **(A)** Ancestry of experimental animals studied that were F1 and F2 generation descendants of animals that were overexposed to thyroid hormone during development (*Dio3−/−* mice, in red). PGM and PGF indicate genetically normal animals with a paternal grandmother or paternal grandfather, respectively, overexposed. **(B)** Expression of the housekeeping gene *Actin b*. **(C)** Expression of T3 responsive genes *Klf9* and *Hr*. Each point represents a different embryo (*n* = 16, 15, 15) and mean ± SEM are shown. Experimental embryos represent 3–4 different litters. ** and *** indicate *P* < 0.01 and 0.001, respectively, as determined by ANOVA and Tukey’s *post-hoc* test.

To determine if these hypomethylated genes exhibit expression changes at earlier stages of brain development in the F2 generation descendants, we use a similar ancestry scheme (Figure 1A) to examine gene expression in embryonic day 13.5 (E13.5) PGF, PGM, and control brains. These experimental fetuses were all genetically normal (wild type) and conceived by wild type mothers (Figure 1A). Expression of *Actb* did not show any significant difference among experimental groups (Figure 1B) as was used as control to correct the expression data of all genes. Considering that our model might epigenetically influence T3 signaling and the expression of T3-responsive genes, first we determined expression levels *Hr* and *Klf9*, two well-established T3 targets, especially in the brain (Chatonnet et al., 2015). We observed no changes in *Hr* expression among experimental groups, while the expression of *Klf9* was significantly elevated in PGF fetal brains compared to controls (Ctrl) and PGM brains (Figure 1C).

**FIGURE 2:**
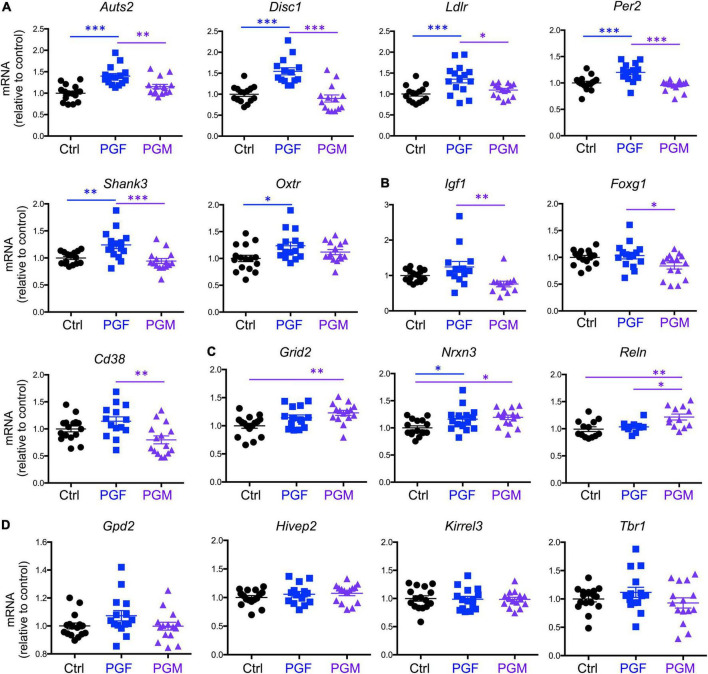
Expression of neurodevelopmental disorders genes in E13.5 PGF and PGM brains. **(A)** Genes showing increased expression in PGF fetal brains. **(B)** Genes showing a decreased in PGM brains. **(C)** Genes showing an increase in PGM or in both PGF and PGM. **(D)** Genes showing no different expression between experimental groups. Each point represents a different embryo (*n* = 16, 15, 15) and mean ± SEM are shown. Experimental embryos represent 3–4 different litters. *, ^**^, and ^***^ indicate *P* < 0.05, 0.01, and 0.001, respectively, as determined by ANOVA and Tukey’s *post-hoc* test.

Then we determined the expression of 16 selected genes that we previously reported as hypomethylated in the sperm of *Dio3−/−* males (Martinez et al., 2020), and that are associated in humans with susceptibility to neurodevelopmental disorders, especially autism, according to the gene compendium maintained by the Simmons Foundation. We observed that the expression of *Auts2*, *Disc1*, *Ldlr*, *Per2*, and *Shank3* was elevated in E13.5 PGF brains, when compared to that in controls and PGM brains, while *Oxtr* expression was increased in PGF brains compared to controls only (Figure 2A). The expression of *Igf1*, *Foxg1*, and *Cd38* showed a different pattern among experimental groups, with their expression significantly reduced in PGM brains (Figure 2B). The expression of *Grid2* and *Nrxn3* was increased in both PGF and PGM embryos, although it did not reach statistical significance for *Grid2* in the PGF group (Figure 2C). The expression of *Reln* was significantly elevated in PGM brains compared to the other two experimental groups (Figure 2C). The expression of four other genes tested, *Gpd2*, *Hivep2*, *Kirrel3*, and *Tbr1* did not show any significant difference between experimental groups (Figure 2D). These data suggest that ancestral overexposure to thyroid hormone can influence brain gene expression of F2 generation descendants as early as E13.5, and that this aberrant expression included genes associated with neurodevelopmental disorders in humans.

### Deficits in social behavior in adult paternal grandfather male mice

**FIGURE 3:**
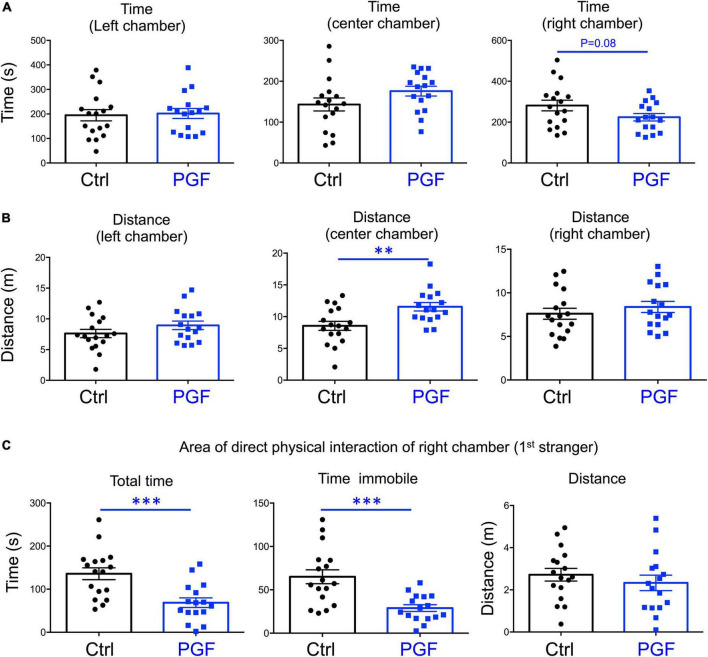
Sociability of PGF males. **(A–C)** Second trial of the three-chamber social box test, after first stranger is introduced in the right chamber. **(A)** Time spent by the test mouse in each chamber. **(B)** Distance traveled by the test mouse in each chamber. **(C)** Total time, time immobile, and distance traveled by the test mouse in the area immediately around the 1st stranger in the right chamber. Each point represents a different mouse tested at approximately 18 weeks of age (*n* = 17, 16) and mean ± SEM are shown. Experimental mice from each group represent 4 different litters and data represent two animal cohorts that were generated and tested at different dates. ^**^ and ^***^ indicate *P* < 0.01 and 0.001, respectively, as determined by the Student’s *t*-test.

In our recent work, we also showed that PGF adult males exhibit decreases in locomotor activity and anxiety-related behavior (Martinez et al., 2020), but sociability, which is altered in directly exposed ancestor mice (Stohn et al., 2018), has not been assessed. Given these observations and the male bias in the prevalence of some neurodevelopmental disorders, here we initially focused on the study of males, and evaluated social behavior in 4-month old PGF and control mice using the three-chamber social box test (Supplementary Figure 1A). During the habituation trial, we observed no significant differences between PGF and control males in the time or distance spent in any of the chambers (Supplementary Figures 1B,C). In the second trial, when the first stranger was placed in the right chamber to assess sociability, PGF males spent less time in the chamber where the first stranger was placed, although this parameter did not reach statistical significance (*P* = 0.08) (Figure 3A). We did not observe differences in distance traveled for right and left chambers, but PGF males covered more distance than controls in the center chamber (Figure 3B), indicating slightly elevated velocity (*P* = 0.09, data not shown) in this chamber. Interestingly, when analyzing the activity in the immediate area surrounding the stranger’s cage, where physical inspection is possible (Supplementary Figure 1A), we observed that PGF males spent significantly less total time and less time immobile than control males (Figure 3C), suggesting diminished interest in exploring the stranger and interacting with it, or increased anxiety about the direct interaction.

**FIGURE 4:**
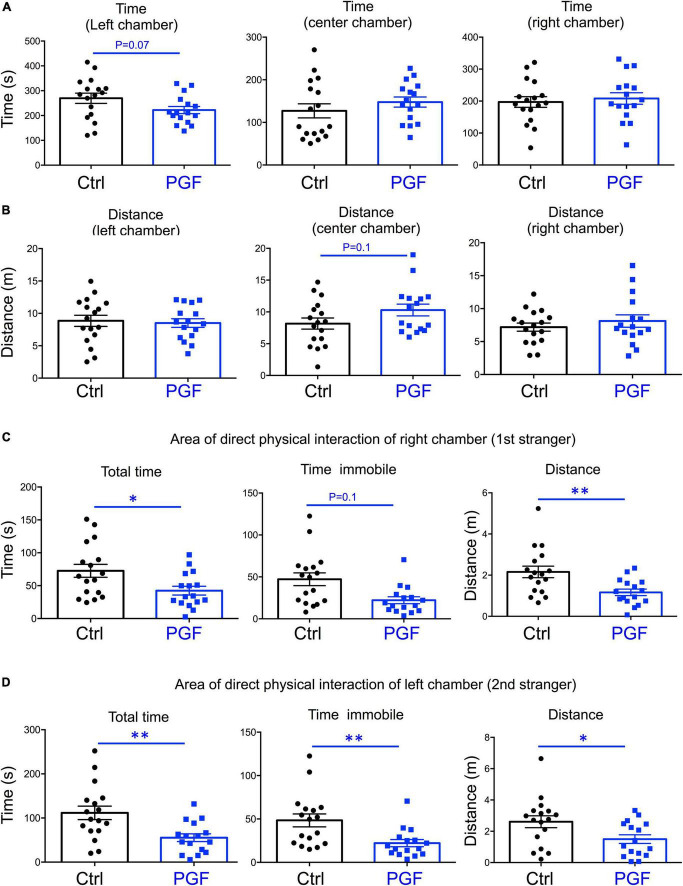
Interest in social novelty of PGF males. **(A–D)** Third trial of the three-chamber social box test after a second stranger is added into the left chamber. **(A)** Time spent by the test mouse in each chamber. **(B)** Distance traveled by the test mouse in each chamber. **(C)** Total time, time immobile and distance traveled by the test mouse in the area immediately around the 1st stranger in the right chamber. **(D)** Total time, time immobile, and distance traveled by the test mouse in the area immediately around the 2nd stranger in the left chamber. Each point represents a different mouse tested at approximately 18 weeks of age (*n* = 17, 16) and mean ± SEM are shown. Experimental mice represent 4–7 different litters and data represent two animal cohorts that were generated and tested at different dates. * and ^**^ indicate *P* < 0.05 and 0.01, respectively, as determined by the Student’s *t*-test.

In the third trial, when a second stranger was added to the left chamber to evaluate interest in social novelty, PGF tended to spend less time in the left chamber (*P* = 0.07) suggesting a decrease in interest for social novelty (Figure 4A). Similarly to the second trial, there was a tendency for PGF males to spend more time and cover more distance in the center chamber, but these increases did not reached statistical significance (Figure 4B). Data for the area of physical interaction with either stranger in the left and right chambers revealed that PGF males spent markedly less total time and time immobile, and traveled less distance than controls, both with first stranger (Figure 4C) and with the novel stranger (Figure 4D). Overall, these results indicate PGF males exhibit decreased tendency for direct social interactions both in sociability and social novelty evaluations.

### Social behavior in F1 generation mice

**FIGURE 5:**
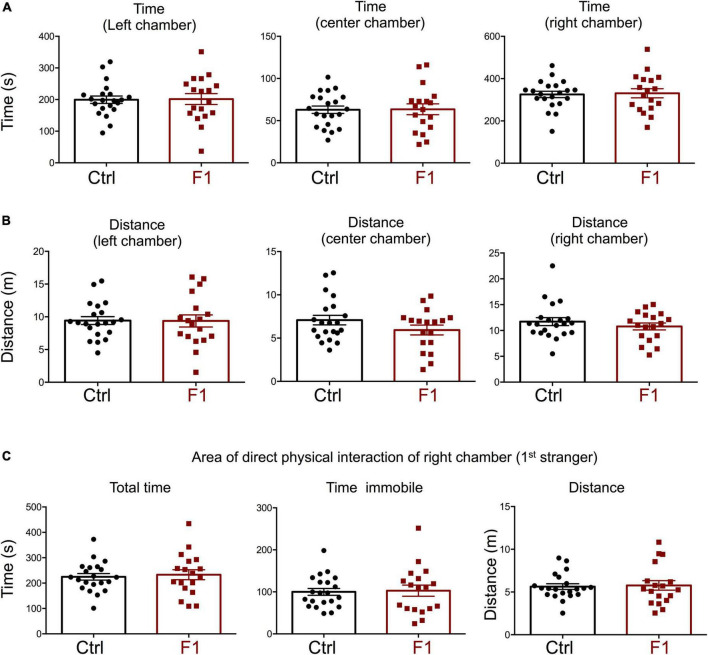
Sociability of F1 generation males. **(A–C)** Second trial of the three-chamber social box test, after first stranger is introduced in the right chamber. **(A)** Time spent by the test mouse in each chamber. **(B)** Distance traveled by the test mouse in each chamber. **(C)** Total time, time immobile, and distance traveled by the test mouse in the area immediately around the 1st stranger in the right chamber. Each point represents a different mouse tested at approximately 18 weeks of age (*n* = 22, 20) and mean ± SEM are shown. Experimental mice from each group represent 4–6 different litters and data represent two animal cohorts that were generated and tested at different dates.

**FIGURE 6:**
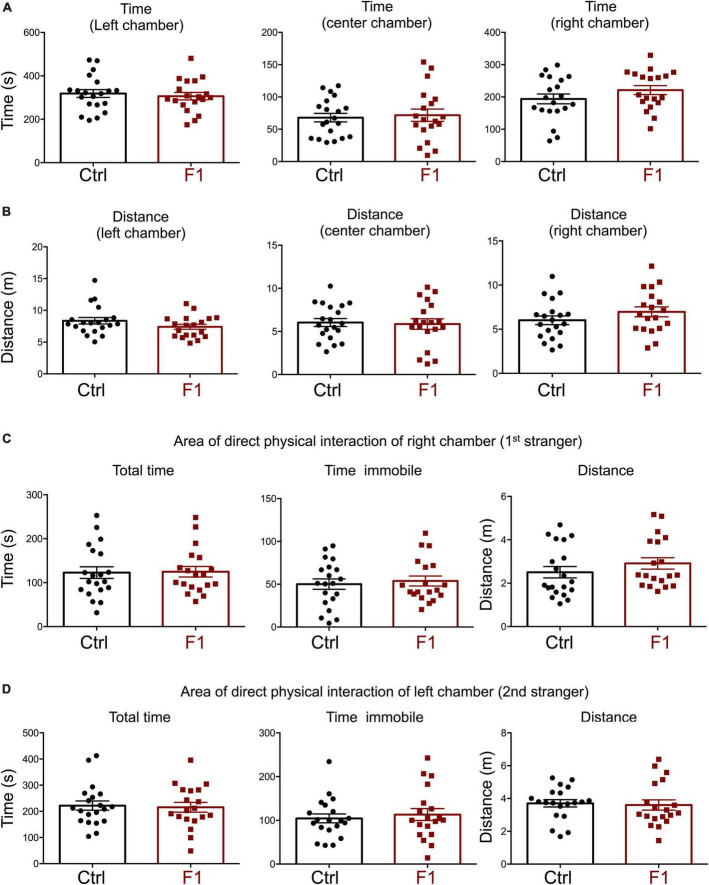
Interest in social novelty of F1 generation males. **(A–C)** Second trial of the three-chamber social box test, after first stranger is introduced in the right chamber. **(A)** Time spent by the test mouse in each chamber. **(B)** Distance traveled by the test mouse in each chamber. **(C)** Total time, time immobile, and distance traveled by the test mouse in the area immediately around the 1st stranger in the right chamber. **(D)** Total time, time immobile, and distance traveled by the test mouse in the area immediately around the 2nd stranger in the left chamber. Each point represents a different mouse tested at approximately 18 weeks of age (*n* = 22, 20) and mean ± SEM are shown. Experimental mice from each group represent 4–6 different litters and data represent two animal cohorts that were generated and tested at different dates.

The deficits in the social behavior of PGF males in the three-chamber social box test is very similar to that in *Dio3−/−* ancestors of both sexes (Stohn et al., 2018), which were directly overexposed to thyroid hormone during development. We then investigated whether these abnormalities in social behavior also exist in the offspring of *Dio3−/−* mice (F1 generation, F1 mice). Since F1 animals generated by *Dio3−/−* males are heterozygous for the *Dio3* mutation, and the mutation was inherited from their fathers (Figure 1A, right side), we used as a control group heterozygous animals that also inherited the *Dio3* mutation from their fathers. However, for this control group, fathers were heterozygous mice that are phenotypically normal, as they were not exposed to any excess of thyroid hormone due to inheritance of the mutation from their mothers (Figure 1A). Surprisingly, adult F1 male mice showed no significant abnormalities in social behavior. Both time spent and distance traveled in each of the chambers were the same as those in control animals during the habituation trial (Supplementary Figure 2), during the second trial (first stranger present, Figures 5A,B), as well as during the third trial (second stranger also present, Figures 6A,B). In addition, we observed no differences in the time spent, time immobile and distance traveled in the area of immediate physical interaction with the strangers during the second trial (Figure 5C) or during the third trial (Figures 6C,D). Interestingly, F1 male mice showed a significant decreased in the number of entries into the chamber of the first stranger during the sociability test, and the entries to the empty chamber were also decreased (Supplementary Figure 3A). During the social novelty trial, with both strangers present, the number of entries to both left and right chambers was also decreased in F1 males (Supplementary Figure 3B), indicating a lower number of longer visits to the social chambers.

**FIGURE 7:**
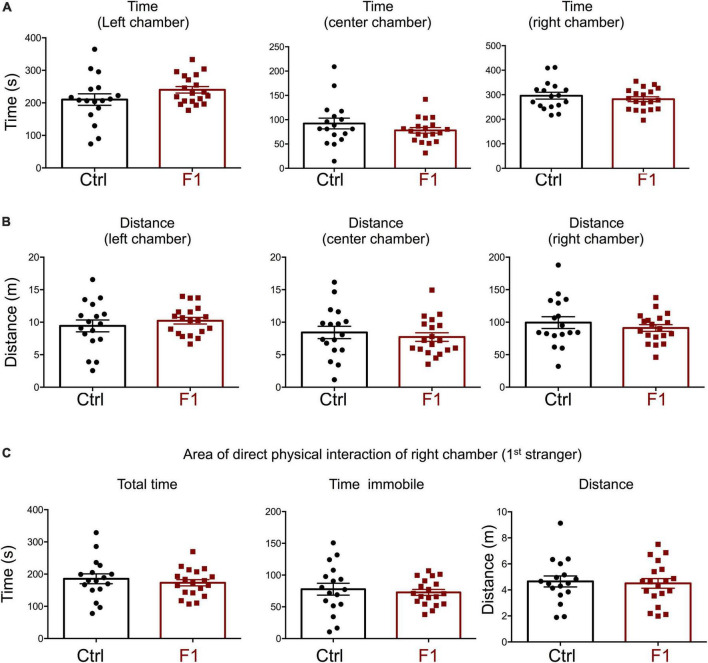
Sociability of F1 generation females. **(A–C)** Second trial of the three-chamber social box test, after first stranger is introduced in the right chamber. **(A)** Time spent by the test mouse in each chamber. **(B)** Distance traveled by the test mouse in each chamber. **(C)** Total time, time immobile, and distance traveled by the test mouse in the area immediately around the 1st stranger in the right chamber. Each point represents a different mouse tested at approximately 18 weeks of age (*n* = 17, 19) and mean ± SEM are shown. Experimental mice from each group represent 4–6 different litters and data represent two animal cohorts that were generated and tested at different dates.

**FIGURE 8:**
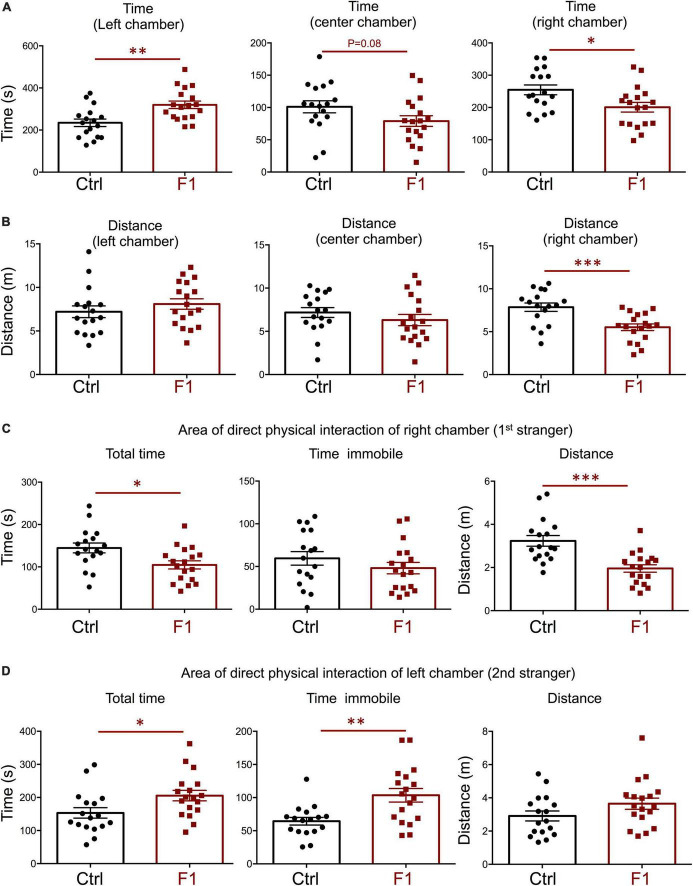
Interest in social novelty of F1 generation females. **(A–C)** Second trial of the three-chamber social box test, after first stranger is introduced in the right chamber. **(A)** Time spent by the test mouse in each chamber. **(B)** Distance traveled by the test mouse in each chamber. **(C)** Total time, time immobile, and distance traveled by the test mouse in the area immediately around the 1st stranger in the right chamber. **(D)** Total time, time immobile, and distance traveled by the test mouse in the area immediately around the 2nd stranger in the left chamber. Each point represents a different mouse tested at approximately 18 weeks of age (*n* = 17, 19) and mean ± SEM are shown. Experimental mice from each group represent 4–6 different litters and data represent two animal cohorts that were generated and tested at different dates. *, ^**^, and ^***^ indicate *P* < 0.05, 0.01, and 0.001, respectively, as determined by the Student’s *t*-test.

In the process of generating the F1 experimental males above, we were also able to produce enough F1 adult females for the assessment of social behavior and determine if the intergenerational epigenetic effects varied with sex. In contrast to F1 and PGF males, F1 females showed a significant preference for the left chamber during the habituation trial (when no strangers are present), as revealed by increased time spent and distance traveled in this chamber (Supplementary Figures 4A,B). During the sociability test, when the first stranger was present, F1 females did not show any difference in the parameters measured. They spent the same time (Figure 7A) and traveled the same distance (Figure 7B) in all chambers as did control females. In addition, F1 females showed no difference in the time spent, time immobile or distance traveled within the area of direct interaction with the first stranger (Figure 7C). However, we observed some significant differences in interest in social novelty, as tested in the third trial. Compared to control females, F1 females spent significantly more time in the chamber of the novel stranger (Figure 8A), and spent less time and traveled less distance in the chamber of the familiar stranger (Figure 8B). Consistent with these parameters, F1 females spent less time, and traveled less distance in the area of direct interaction with the familiar stranger (Figure 8C), and spent significantly more time than female controls in the center chamber, as well as in the chamber of the novel stranger (Figure 8D). These results suggest F1 females have increased interest in social novelty.

### Fetal brain gene expression in F1 generation mice

**FIGURE 9:**
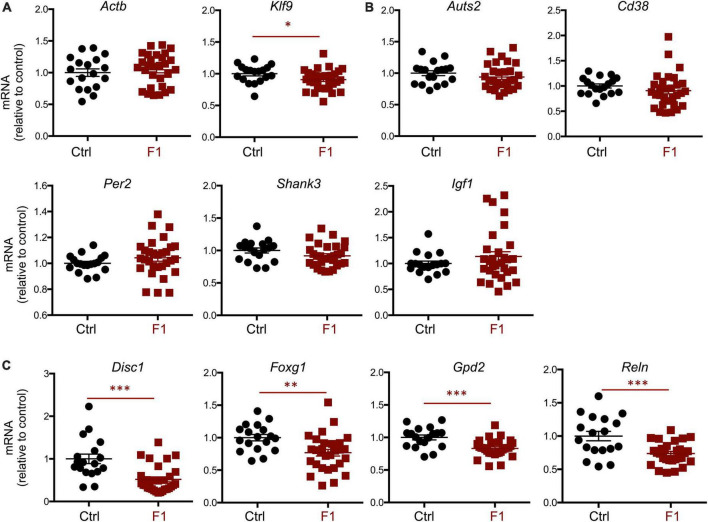
Gene expression in F1 generation E13.5 brains. **(A)** Expression of house-keeping gene *Gapdh* and thyroid hormone-responsive *Klf9*. **(B)** Genes showing no significant difference in expression. **(C)** Genes showing a significant decrease in expression. Each point represents a different embryo (*n* = 18, 29) and mean ± SEM are shown. Experimental embryos represent 3–4 different litters. *, ^**^, and ^***^ indicate *P* < 0.05, 0.01, and 0.001, respectively, as determined by the Student’s *t*-test.

Given the differences in social behavior between F1 and PGF males, we generated E13.5 F1 fetuses to examine the expression of neurodevelopmental genes that exhibited abnormal expression in the fetal brain of the F2 generation (PGF fetuses, Figure 2). Contrary to PGF fetuses, F1 fetuses exhibited a modest but significant decrease in the expression of thyroid hormone-sensitive gene *Klf9* (Figure 9A), suggesting a mildly altered thyroid status that is opposite to that in PGF fetal brain. Some genes that showed altered expression in the brain of PGF and/or PGM fetuses, did not show altered expression levels in F1 fetal brains. These genes included *Auts2*, *Cd38*, *Per2*, *Shank3*, and *Igf1* (Figure 9B). However, F1 fetuses showed decreased expression of some autism-susceptibility genes, including *Disc1*, *Foxg1*, *Gdp2*, and *Reln* (Figure 9C), indicating that aberrant brain expression of some ASD-related genes also occurs in the F1 generation. Considering the difference in social behavior parameters between F1 males and females, we explored if sexual differences were also observed in fetal gene expression. Segregation according to sex of brain expression data on genes differentially expressed in F1 fetuses revealed that the main determinant of expression difference was ancestry, as no significant differences in gene expression were observed between males and females (Supplementary Figure 5A). Similar analyses of the data shown in Figure 2 concerning differentially expressed genes in the brain of PGF and PGM fetuses also indicated no effect of sex (Supplementary Figure 5B). In addition, the expression of thyroid hormone receptor *Thra* [most abundant at this stage (Martinez and Hernandez, 2021)] and *Thrb* was not significantly different in the brain of F1 (Supplementary Figure 6A) or PGF and PGM fetuses (Supplementary Figure 6B), suggesting that they are not mediating the altered gene expression in descendants.

Of note, the change in methylation previously described in the ancestral sperm (Martinez et al., 2020) did not necessarily show an inverse correlation with the expression changes in the brain of F1 and PGF fetuses, suggesting that for some genes the sperm methylation status in exposed ancestors is not directly inherited in subsequent generations.

## Discussion

Many complex conditions in humans, especially neurological disorders, exhibit high heritability, but candidate genes identified in GWAS have come significantly short of explaining the actual number of clinical cases (Manolio et al., 2009; Escher and Ford, 2020). In this regard, epigenetic-based effects across generations elicited by environmental conditions in ancestors provide a potential mechanism contributing to the onset of disease in genetically normal or already susceptible individuals. Thyroid hormones are among these factors, and studies in mice (Martinez et al., 2020), rats (Bakke et al., 1976, 1977), and humans (Anselmo et al., 2019) have shown that alterations in ancestral thyroid hormone status can cause phenotypic effects in subsequent generations. In addition, epidemiological studies in humans have shown that parental alterations in thyroid hormone status and physiology may influence children’s susceptibility to autism (Molloy et al., 2006; Sadamatsu et al., 2006; Andersen et al., 2014; Khan et al., 2014), schizophrenia (Gyllenberg, 2016), and ADHD (Andersen et al., 2014; Modesto et al., 2015).

Using a genetic mouse model of developmental thyrotoxicosis due to a lack of thyroid hormone clearance (*Dio3−/−* mouse), we have recently shown abnormalities in locomotor activity and anxiety-related behavior in genetically normal F2 generation male descendants of thyroid hormone overexposed mice of either sex (Martinez et al., 2020). These abnormal behaviors were associated with altered gene expression profiles in several brain regions at postnatal day 15. However, differentially expressed genes at this particular age did not sufficiently coincide with differentially methylated genes in the sperm of ancestors overexposed to thyroid hormones (Martinez et al., 2020).

To determine if these genes are differentially expressed in the brain of descendants at much earlier stages of development, we investigated the brains of F1- and F2 generation descendants at E13.5 and measure the expression of selected autism-related genes that were differentially methylated in the sperm of thyroid hormone overexposed ancestors (Martinez et al., 2020). We observed that the expression of many of these genes is altered in fetuses that had a paternal grandmother or grandfather overexposed to T3. Altered gene expression in some of these genes is also evident in the fetal brains of the immediate offspring (F1 generation) of T3-overeposed males. However, despite some overlapping, these changes were not necessarily the same in the F1 and F2 generation descendants. Changes in PGF and PGM fetuses (F2 generation) did not always coincide or show the same trend. These observations suggest that the sex of the T3-overexposed ancestor may also influence the epigenetic changes passed to the next generation. In addition, gene methylation differences previously described in the sperm of exposed ancestors (Martinez et al., 2020) did not necessarily predict the gene expression changes in the brain of F1 and PGF fetuses, suggesting that some intergenerational effects may result from secondary effectors. Based on our data, these secondary effectors do not appear to include abnormal thyroid hormone receptor abundance. It is also possible that some of the intergenerational effects on brain gene expression have an allele-specific origin, or result from *in trans* epigenetic cross-talk between alleles. Still, for some genes, methylation marks established in the sperm of overexposed animals may be maintained in the brain of descendants, and this hypothesis will be tested in future studies. Overall, our results also support the hypothesis that some of the epigenetic modifications mediating the phenotypic effects on fetal brain gene expression may be reversed and re-established across generations. In this regard, we have previously shown that DIO3 is located in spermatogonia and that DIO3-deficiency profoundly affects gene expression in the neonatal testis (Ref). The vast majority of down regulated genes included most histone genes and were enriched in functions related to chromatin organization and nucleosome structure (Martinez et al., 2019), providing a mechanism by which thyroid hormone excess impacts the epigenetic information of the germ line.

This idea is also consistent with the social behavior deficits of F2 generation males in the social box test. Their significant reduced time and stillness in the area of physical interaction with the strangers recapitulates the behavioral phenotype of the F0 male ancestors originally overexposed to T3 (Stohn et al., 2018). However, this social deficit phenotype is not observed in the F1 generation, again suggesting that some neurological phenotypes associated with epigenetic effects of thyroid hormone may skip a generation, and appear every other generation. This is an intriguing observation and may result from the fact that the F1 generation animals studied are not genetically normal as those studied in the F2 generation. It is thus possible that the abnormal epigenetic information inherited by F1 mice does not exert the same effect in a genetically abnormal mouse, pointing to the *Dio3* genomic region as a potential modulator of some of the intergenerational epigenetic effects.

In contrast, other behavioral traits may be maintained, reversed or normalized across generation in this model, as we have previously reported the same anxiety-related phenotype, but opposite physical activity phenotype in F0 exposed males compared to PGF males (Martinez et al., 2020). Although we did not observe evidence of low physical activity of PGF males in the social behavior test, this may be due to the time limitation and different social environment of this test compared with a 48 h monitoring in metabolic cages (Martinez et al., 2020).

Still, despite the apparent absence of social deficits in F1 males, we recorded a significantly lower number of entries in social chambers for F1 males, both in the sociability and social novelty trials of the social box paradigm. All other parameters being equal, this observation indicates their visits to the strangers’ chambers were fewer and longer, suggesting less restlessness in the face of potential social interaction and a subtle increase in sociability. Interestingly, initial results in F1 females indicate no changes in sociability, but a significantly elevated interest in social novelty, as determined by the less time spent with the first stranger when the second was also placed in the social box. These increased social novelty interest in F1 females but not males, suggest that the sex of the affected individual also contributes to the generation of specific neurological traits as a result of the intergenerational epigenetic effects elicited by thyroid hormone. This sex differences are also consistent with the sex bias and sexually dimorphic characteristics of human neurodevelopmental disorders characterized by social deficits (Skuse, 2000; Baron-Cohen et al., 2005; Knickmeyer and Baron-Cohen, 2006; Hu et al., 2021). However, as we observed no sex differences in the altered gene expression of F1 mice brains (nor in PGF or PGM brains), we speculate that the same epigenetic abnormalities in fetal gene expression may impact males and females in a different manner later in development, probably during the period of neonatal brain sexual differentiation.

As thyroid hormones have a profound impact on brain development, and we used a model of thyroid hormone overexposure, it is possible altered levels of thyroid hormone signaling are part of the intergenerational effects on descendants. Although the fetal brain in rodents has long been considered largely insensitive to thyroid hormones (Schwartz et al., 1997; Grijota-Martínez et al., 2011), we have recently shown that this is not the case, and that the E13.5 brain may show increased expression of thyroid hormone responsive genes (e.g., *Klf9*) when thyroid hormone clearance is lost (i.e., in the *Dio3−/−* mouse) (Martinez and Hernandez, 2021). In our present experiments, the expression of T3-regulated *Klf9* is significantly altered in the brain of F1 and PGF fetuses, suggesting abnormal levels of thyroid hormone action. The fact that *Klf9* expression is decreased in F1 but increased in PGF fetal brains correlates with the observed differences between generations in social behavior and fetal brain gene expression patterns, further suggesting that the original phenotype observed in thyroid hormone overexposed ancestors is partially recapitulated in F2 generation descendants across the paternal lineage, but largely skips the F1 generation.

Our data shows intergenerational epigenetic effects of thyroid hormone on social behavior and developmental brain gene expression, but future investigations are needed to ascertain which specific alterations in gene expression, and which ancestry patterns of exposure, can predict the epigenetic inheritance of neurological phenotypes and susceptibility to deficits in social behavior. Genetic studies in humans indicate that either deficiencies (caused by mutations) (Brandler et al., 2016; Lammert and Howell, 2016; Mercer et al., 2016; Onay et al., 2016; Yuen et al., 2016), or increases (elevated gene copy number) (Sebat et al., 2007; Bucan et al., 2009; Glessner et al., 2009; Pinto et al., 2010; Guo et al., 2017), in ASD-candidate genes can be associated with the condition. This suggests that the underlying biological processes affected during central nervous system development can be similarly disrupted by both increases or decreases in relevant gene expression, and that normal outcomes require precisely timed gene expression levels. This notion is also illustrated by the effect of thyroid hormone on the perinatal development of the auditory system. Too much hormone, too early (due to impaired thyroid hormone clearance), or too little, too late (due to loss of timely cochlear generation of the active hormone, T3, from thyroxine), leads to cochlear defects and deafness (Ng et al., 2004, 2009).

Additional work with these paradigms of ancestral thyroid hormone exposure needs to be performed to fully understand how gene expression alterations in the fetal and adult central nervous system correlates with specific social behavior phenotypes, and with particular germ line and brain cell epigenetic signatures and transcriptomic profiles. How ancestry lineage and the sex of both the exposed animal and the descendants exert differential effects on the neurobehavioral traits of descendants needs to be discerned. Our present work demonstrates that ancestral alterations in thyroid hormone exposure can influence social behavior in subsequent generations, as well as fetal brain expression profiles of ASD-candidate genes that could influence susceptibility to neurological conditions. As thyroid disease is relatively common in humans, these findings may increase our considerations of the potential factors contributing to the non-genetic—but heriTables—etiology of prevalent neurodevelopmental conditions, including autism and schizophrenia.

## Data availability statement

The raw data supporting the conclusions of this article will be made available by the authors, without undue reservation.

## Ethics statement

This animal study was reviewed and approved by MaineHealth Institute for Research Institutional Animal Care and Use Committee.

## Author contributions

AH designed the studies and drafted the manuscript. MM, JS, EM, and RW generated experimental animals and data and drafted results. All authors contributed to the editing of the manuscript and approved the submitted version.
