# Supplementary material for: Thyroid hormone elicits intergenerational epigenetic effects on adult social behavior and fetal brain expression of autism susceptibility genes
Source: Front Neurosci. 2022 Nov 7;16:1055116. doi: 10.3389/fnins.2022.1055116 (PMC9676973; doi:10.3389/fnins.2022.1055116)
Supplement: Supplementary file 1 [file Presentation_1.pdf]

Supplementary Table 1. Sequences of primers used in qPCR

Gene Symbol	Forward primer	Reverse primer
Actb	TGGGTATGGAATCCTGTGGC	CTGCATCCTGTCAGCAATGC
Klf9	GGCTGTGGGAAAGTCTATGG	AAGGGCCGTTCACCTGTATG
Hr	AGC ACT GTG TGG CAT GTG TT	AAC CCT GCA TCC AAG TAG CA
Auts2	GGAGGTCTCGATCACAGCG	TTCGGCTGAGGTGGACTCT
Disc1	GCACTTTGCGGTTCATTCCAA	GGAGCCAGAGACTTAAAGCTG
Ldlr	TGACTCAGACGAACAAGGCTG	ATCTAGGCAATCTCGGTCTCC
Per2	CCTTCAGACTCATGATGACAGAGGCAGA	GGCCTTCTTGTCTGCAGGGAGGT
Shank3	CCGGACCTGCAACAAACGA	GCGCGTCTTGAAGGCTATGAT
Oxtr	TTCTTCGTGCAGATGTGGAG	ACGAGTTCGTGGAAGAGATG
Igf1	TGAGCTGGTGGATGCTCTT	CACTCATCCACAATGCCTGT
Foxg1	AGCGACGACGTGTTCATCG	CCCGTTGTAACTCAAAGTGCTG
Cd38	GCCTGCGTGGATAACTACAG	GTGCTCAGGGTTCTTCACAC
Grid2	AACACGCTACATGGACTACTC	GAAGCACTGTGCCAGCAATG
Nrxn3	TGTCTACCACCGTCATGGAAA	GGTCTAAAGTCCTTGGATTCCCA
Reln	CAAGCACCTTCTTTGATGGC	GTAGGCAGGTGACTCACATG
Gpd2	CACTAGATGCCGTCACCAGAG	GAAGGGCTTCTTTCACCATCC
Hivep2	GGGGACACAGCTCTAGGACAA	GCCCTTCCTGACTGCTGAAAG
Kirrel3	GGCTTCGTCCTGTGGATCAAA	CTGGCACTCATACACGGCAT
Tbr1	GGTTGGTGCCCGGCAAGGC	GAGCGGTGGGATCGAGACCAGA

Supplementary Table 2. Gene sperm methylation and expression in descendants

Gene	Methylation in F0 sperm	Expression in F1 fetal brains	Expression in PGF fetal brains
Auts2	Hypomethylated	Unchanged	Elevated
Disc1	Hypo / hypermethylated	Reduced	Elevated
Ldlr	Hypomethylated	ND	Elevated
Per2	Hypo / hypermethylated	Unchanged	Elevated
Shank3	Hypomethylated	Unchanged	Elevated
Oxtr	Hypomethylated	ND	Elevated
Igf1	Hypo / hypermethylated	Unchanged	Unchanged
Foxg1	Hypomethylated	Reduced	Unchanged
Cd38	Hypo / hypermethylated	Unchanged	Unchanged
Grid2	Hypomethylated	ND	Unchanged
Nrxn3	Hypomethylated	ND	Elevated
Reln	Hypomethylated	Reduced	Unchanged
Gpd2	Hypo / hypermethylated	Reduced	Unchanged
Hivep2	Hypo / hypermethylated	ND	Unchanged
Kirrel3	Hypo / hypermethylated	ND	Unchanged
Tbr1	Hypomethylated	ND	Unchanged

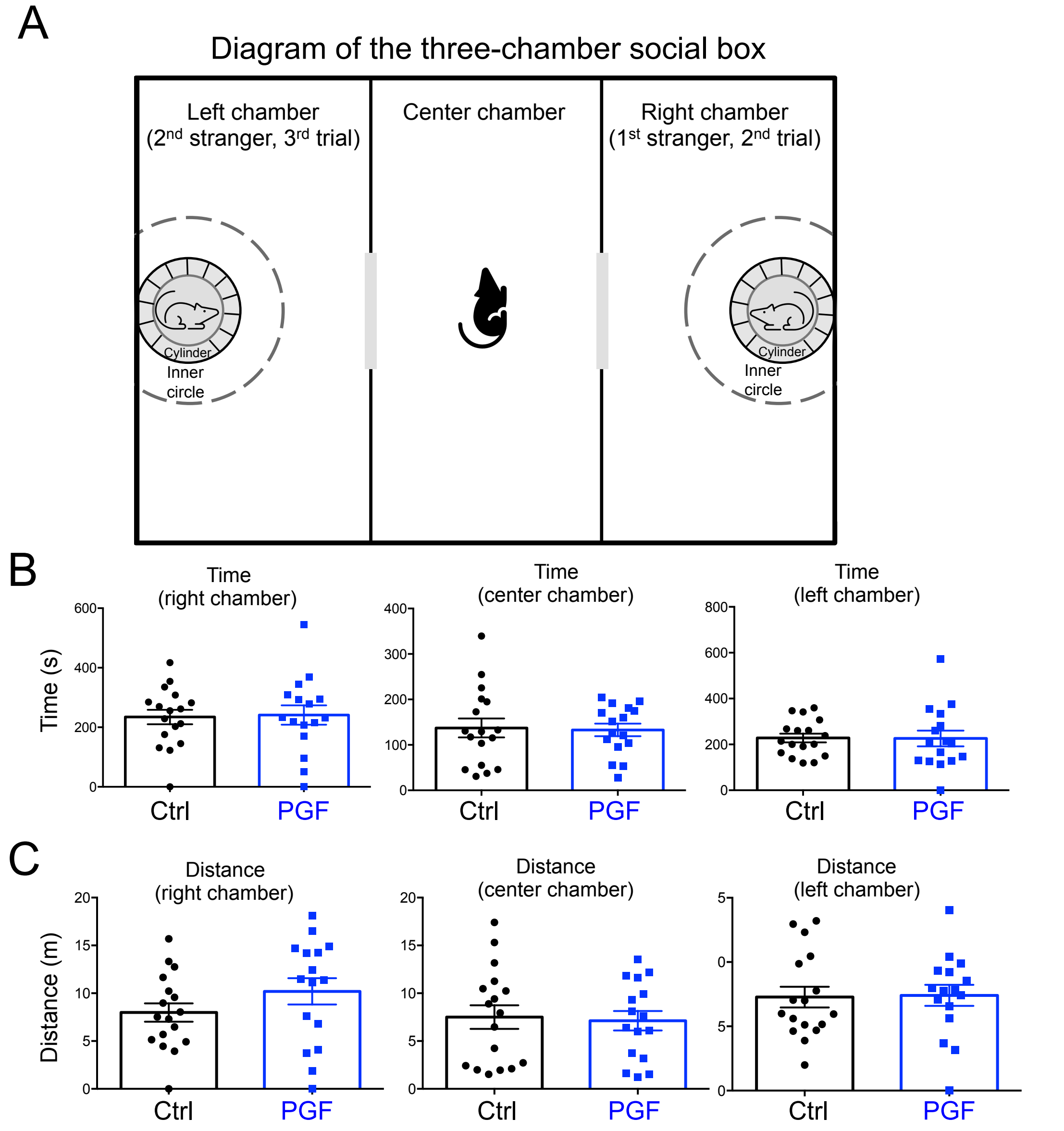

Supplementary Figure 1. Three-chamber social box test and habituation trial in PGF males (first trial). A, On scale diagram of the three-chamber social box test. B and C, Time spent (B) and distance traveled (C) in each of the chambers during the habituation trial (first trial). Each point represents a different mouse tested at approximately 18 weeks of age (n= 17, 16) and mean ± SEM are shown. Experimental mice from each group represent 4 different litters and data represent two animal cohorts that were generated and tested at different dates.

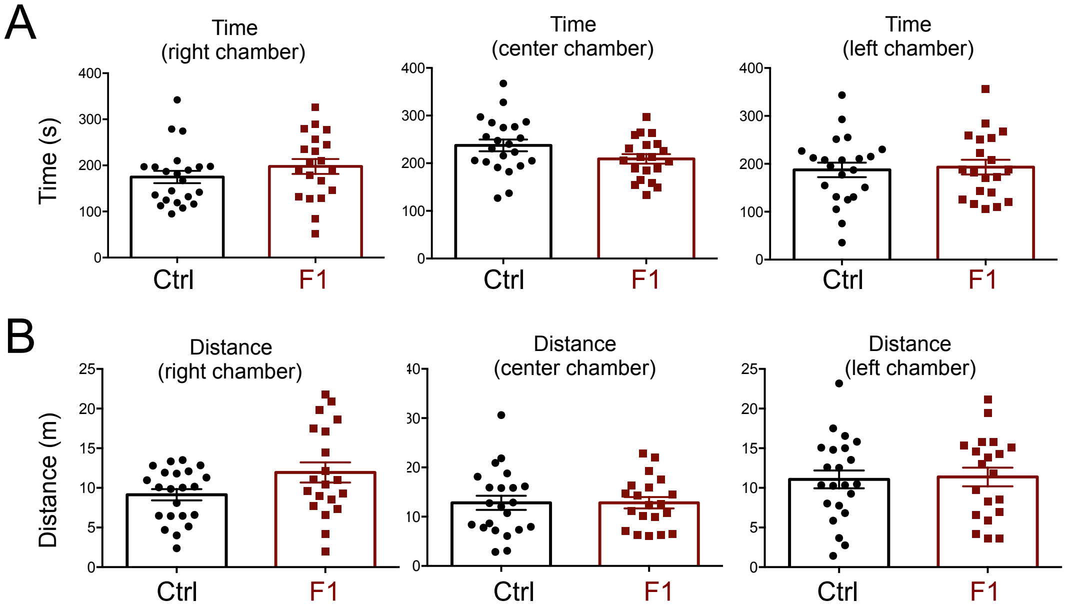

Supplementary Figure 2. Three-chamber social box test and habituation trial in F1 males (first trial). A and B, Time spent (A) and distance traveled (B) in each of the chambers during the habituation trial (first trial). Each point represents a different mouse tested at approximately 18 weeks of age (n= 20, 22) and mean ± SEM are shown. Experimental mice from each group represent 4-6 different litters and data represent two animal cohorts that were generated and tested at different dates.

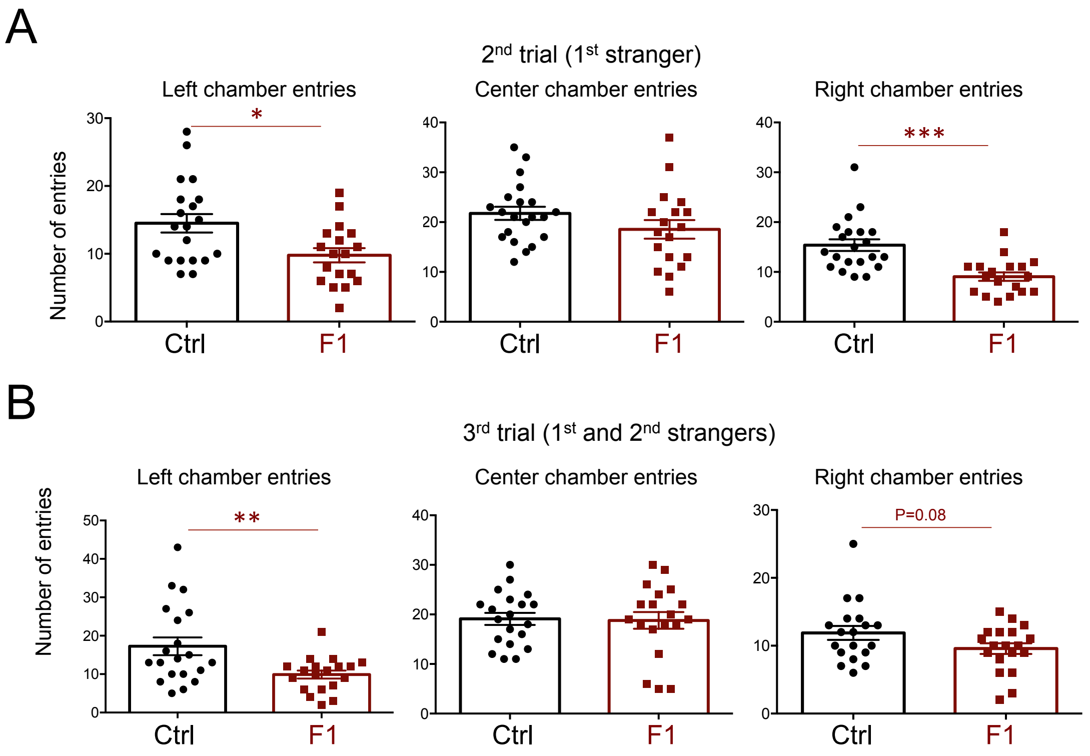

Supplementary Figure 3. Chamber entries of F1 males in the three-chamber social box test during the second and third trial (one and two strangers present, respectively). A and B, Number of entries into each chamber during the second (A) or the third (B) trials. Each point represents a different mouse tested at approximately 18 weeks of age (n= 20, 22) and mean ± SEM are shown. Experimental mice from each group represent 4-6 different litters and data represent two animal cohorts that were generated and tested at different dates. *, ** and ***, P<0.05, 0.01 and 0.001, respectively, as determined by the Student's t-test.

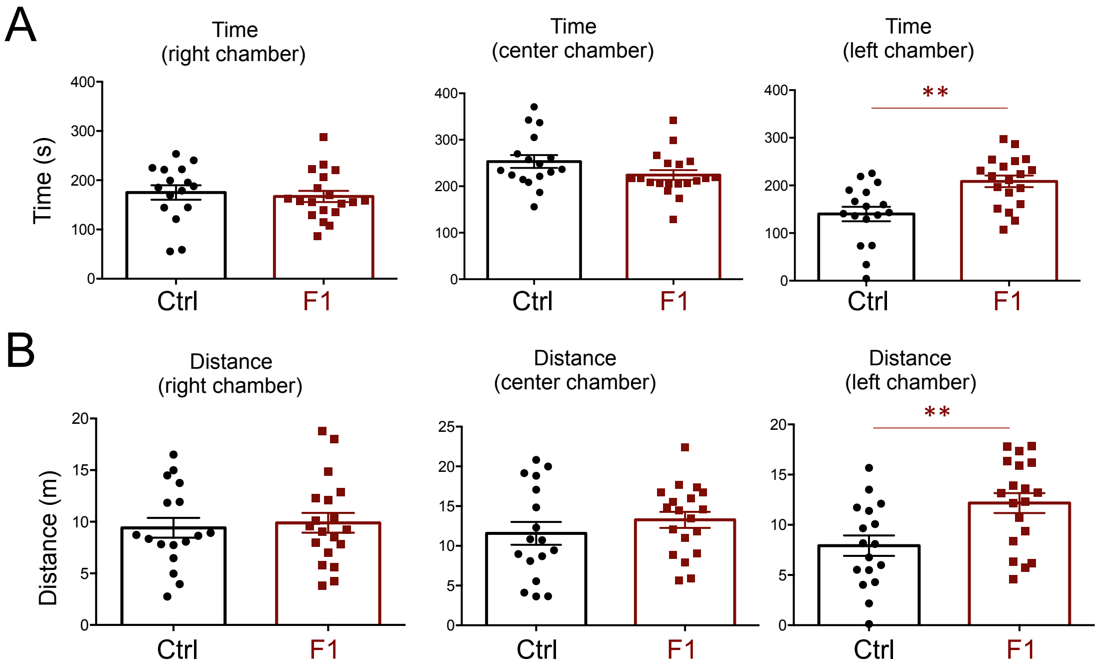

Supplementary Figure 4. Three-chamber social box test and habituation trial in F1 females (first trial). A and B, Time spent (A) and distance traveled (B) in each of the chambers during the habituation trial (first trial). Each point represents a different mouse tested at approximately 18 weeks of age (n= 17, 19) and mean ± SEM are shown. Experimental mice from each group represent 4-6 different litters and data represent two animal cohorts that were generated and tested at different dates. **, P<0.01 as determined by the Student’s t-test.

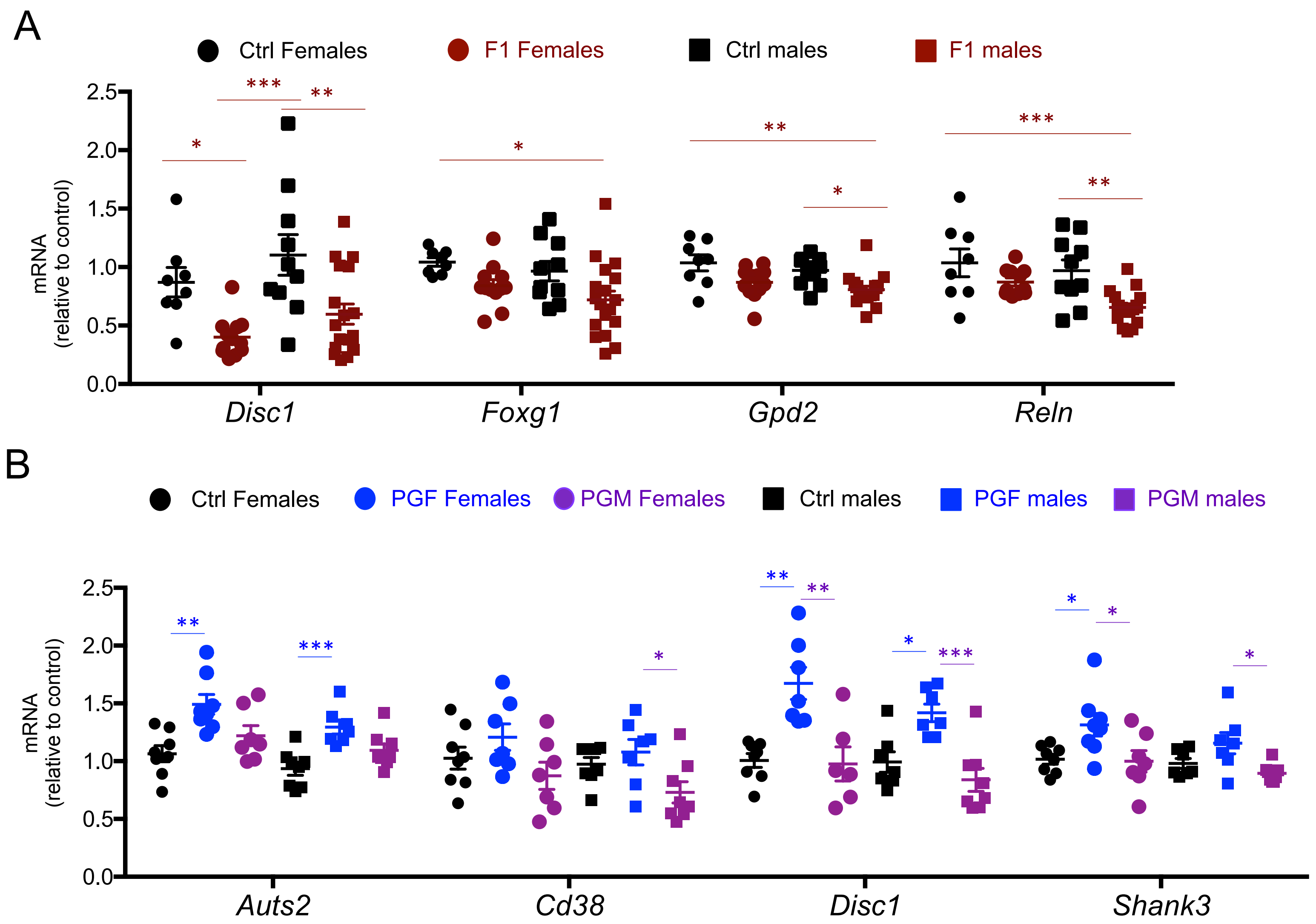

Supplementary Figure 5. No apparent effect of sex on differential brain gene expression in F1- and F2-generation fetuses. A, Data on differentially expressed genes in the F1 generation separated by sex. B, Data on some differentially expressed genes in PGF and PGM fetuses separated by sex. Each point represents a different fetus (n= 8, 12, 10, 18) (A) (n=8, 8, 7, 8, 7, 8) (B). Mean ± SEM are shown. For each experimental group mice represent 3-4 different litters. *, **, *** P<0.05, 0.01, 0.001, respectively, as determined by ANOVA and Tukey's post hoc test.

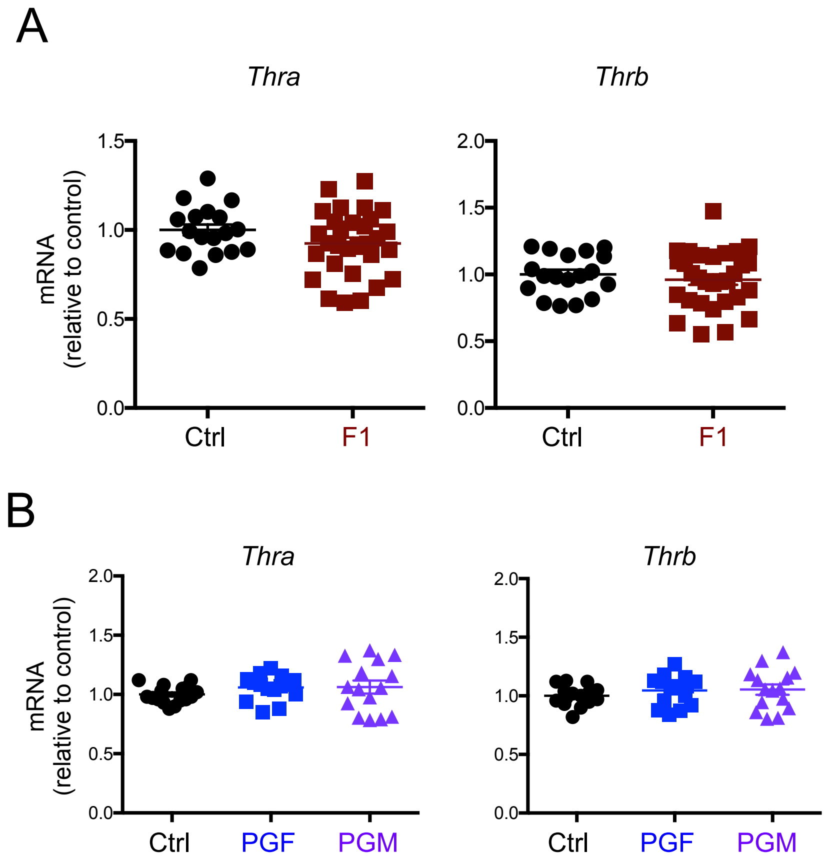

Supplementary Figure 6. Expression of thyroid hormone receptor genes in F1- and F2-generation fetal brains. A, Thyroid hormone receptor expression in the F1 generation fetal brain. B, Thyroid hormone receptor expression in the PGF and PGM fetal brain Data on some differentially expressed genes in PGF and PGM fetuses separated by sex. Each point represents a different fetus (n=18, 30) (A)(n=16, 15, 15)(B). Mean ± SEM are shown. For each experimental group mice represent 3-4 different litters. **, P<0.01 as determined by ANOVA and Tukey's post hoc test.
